# Formative acceptance of ingestible biosensors to measure adherence to TB medications

**DOI:** 10.1186/s12879-022-07756-x

**Published:** 2022-09-28

**Authors:** Clint Vaz, Nisha K. Jose, Jeremiah Jacob Tom, Georgia R. Goodman, Jasper S. Lee, Rana Prathap Padappayil, Manjunath Madathil, Conall O’Cleirigh, Rashmi Rodrigues, Peter R. Chai

**Affiliations:** 1 Department of Emergency Medicine, Brigham and Women’s Hospital, 75 Francis Street, Boston, MA 02114 USA; 2 Department of Psychosocial Oncology and Palliative Care, Dana Farber Cancer Institute, Boston, MA USA; 3 Division of Non-Communicable Diseases, Indian Council of Medical Research, HQs, New Delhi, India; 4 Department of Gastroenterology, Amala Institute of Medical Sciences, Thrissur, Kerala India; 5 The Fenway Institute, Fenway Health, Boston, MA USA; 6 Department of Psychiatry, Massachusetts General Hospital, Boston, MA USA; 7 Department of Internal Medicine, Monmouth Medical Center, Long Branch, NJ USA; 8 Department of Dermatology, Amala Institute of Medical Sciences, Thrissur, Kerala India; 9 Department of Dermatology, Imbichibava Memorial Co-Operative Hospital and Research Center, Malappuram, Kerala India; 10 Department of Community Health, St. John’s Medical College, St. John’s Research Institute, Bangalore, India; 11 Koch Institute for Integrated Cancer Research, Massachusetts Institute of Technology, Cambridge, MA USA

**Keywords:** Tuberculosis, Antitubercular therapy, Adherence, Digital pill system, Ingestible sensors

## Abstract

**Background:**

Tuberculosis (TB) represents a significant public health threat in India. Adherence to antitubercular therapy (ATT) is the key to reducing the burden of this infectious disease. Suboptimal adherence to ATT and lack of demonstrated feasibility of current strategies for monitoring ATT adherence highlights the need for alternative adherence monitoring systems.

**Methods:**

A quantitative survey was conducted to assess the acceptance of and willingness to use a digital pill system (DPS) as a tool for monitoring ATT adherence among stakeholders directly involved in the management of patients with TB in India. Participants reviewed a video explaining the DPS and completed a survey, which covered sociodemographics, degree of involvement with TB patients, initial impressions of the DPS, and perceived challenges for deploying the technology in India. Participants were also asked to interpret mock DPS adherence data.

**Results:**

The mean age was 34.3 (SD = 7.3), and participants (N = 50) were predominantly male (70%). The sample comprised internists (52%) and pulmonologists (30%), with a median of 4 years’ experience (IQR 3, 6) in the management of TB patients. No participants had previously used a DPS, but some reported prior awareness of the technology (22%). Most reported that they would recommend use of a DPS to patients on ATT (76%), and that they would use a DPS in both the intensive and continuation phases of TB management (64%). The majority viewed the DPS (82%) as a useful alternative to directly observed therapy-short course (DOTS), particularly given the ongoing COVID-19 pandemic. Participants reported that a DPS would be most effective in patients at risk of nonadherence (64%), as well as those with past nonadherence (64%). Perceived barriers to DPS implementation included lack of patient willingness (92%), cost (86%), and infrastructure constraints (66%). The majority of participants were able to accurately interpret patterns of adherence (80%), suboptimal adherence (90%), and frank nonadherence (82%) when provided with mock DPS data.

**Conclusions:**

DPS are viewed as an acceptable, feasible, and useful technology for monitoring ATT adherence by stakeholders directly involved in TB management. Future investigations should explore patient acceptance of DPS and pilot demonstration of the system in the TB context.

**Supplementary Information:**

The online version contains supplementary material available at 10.1186/s12879-022-07756-x.

## Background

Tuberculosis (TB) continues to represent a significant public health problem worldwide. According to the United States (US) Centers for Disease Control and Prevention (CDC), an estimated 2 billion people are infected with TB, and 1.5 million deaths are caused by TB each year. In 2020, 9.9 million new cases of TB were reported globally [1]. In response to the substantial global burden of disease, the World Health Organization (WHO) has set an ambitious target of an 80% reduction in new cases and 90% reduction in TB deaths by 2030 as part of their sustainable development goals [1, 2]. India has the highest incidence of TB (26%) in the world, with significant rates of TB and HIV co-infection, as well as multidrug-resistant TB (MDR-TB) (27%) [2, 3]. Suboptimal treatment of TB involving early cessation or imperfect adherence to antitubercular treatment (ATT) has resulted in the development of MDR-TB, continued disease transmission, and persistent endemic TB across the country [3]. Because of the central role that adherence to ATT plays in the eradication of TB, there is an increased focus on advancing strategies for measuring patients’ adherence to ATT in India [4].

One key strategy in TB management centers around measuring ATT adherence over time and responding to episodes of nonadherence [5]. The goal of this approach is to mitigate nonadherence as it occurs to prevent the development of drug resistance and disease transmission. In India, current initiation of ATT during the intensive phase (i.e., first 2 months of treatment after a TB diagnosis) is completed via in-person, directly observed therapy-short course (DOTS) or 99DOTS (a mobile phone-modified DOTS), in concordance with the WHO’s End TB strategy [6]. Despite widespread implementation of DOTS, data from 2013 showed that only an estimated 45% of patients were adherent or completed the entire course of ATT treatment [7].

Given the suboptimal results found with the DOTS approach, the Revised National Tuberculosis Programme (RNTCP) launched 99DOTS – a mobile phone-based strategy for remote adherence monitoring, which utilizes prepackaged ATT and displays unique phone numbers for patients to call after each daily ingestion– as a supplement to in-person DOT. Since its launch in 2015, the efficacy of 99DOTS has been variable across the country [8–11]. Given the low specificity and sensitivity of accurate adherence measurement via DOTS and 99DOTS, other systems have been explored in India as adjunct strategies or replacements, including video DOT (VDOT) and smart pill caps (e.g., medication event monitor systems) [11–13]. VDOT mimics conventional DOTS but involves the additional use of video technology to observe ATT ingestions, which has raised concerns surrounding personal privacy, poor wireless infrastructure, network connectivity issues, and the need to observe each pill ingestion [11, 13]. The Medication Event Reminder Monitor (MERM) system comprises a digital pillbox that uses visual and audio-based cues to remind patients to take their medication; however, MERM risks over-reporting or under-reporting, similar to 99DOTS, as both systems depend primarily on patient behavior; these tools may be most useful as supportive tools for adherence management, as they lack the objectivity of directly observed ingestions [14]. In addition, the MERM system’s large size and audiovisual cues may cause concerns around potential disease disclosure for some patients [9]. Therefore, an alternative adherence monitoring system that is accurate, patient-centered, and convenient for healthcare providers to monitor adherence, is critically needed.

**Fig. 1:**
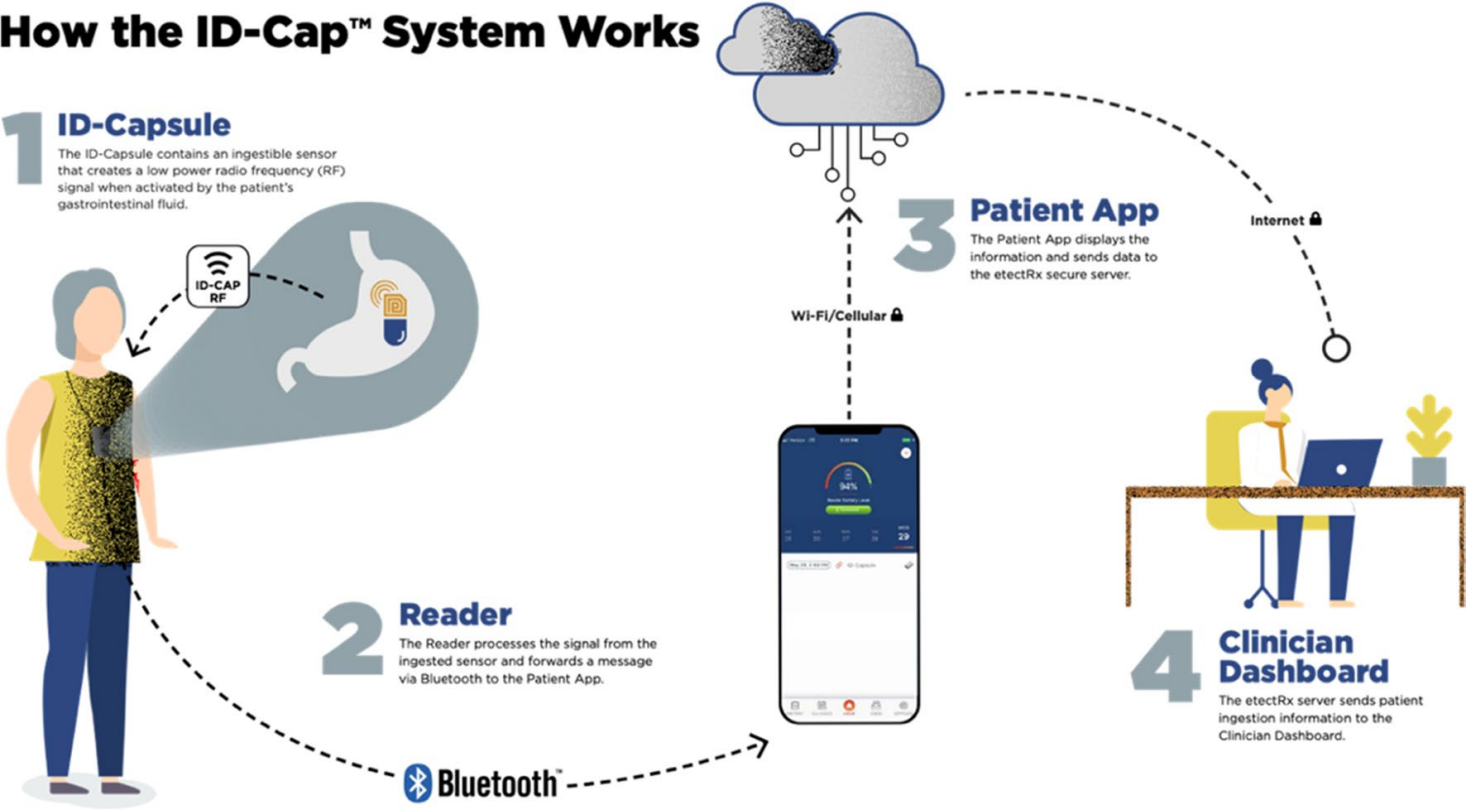
Overall functionality of the US Food and Drug Administration (FDA)-cleared etectRx ID-Cap System, the digital pill system (DPS) utilized in this study. Image courtesy of etectRx

In response to the continued challenge of directly measuring and promoting ATT adherence, multiple technological systems have been developed to provide indirect, surrogate measures of ingestion or direct confirmation of adherence in a remote context. One such technology, the digital pill system (DPS), comprises a radiofrequency identification (RFID)-tagged gelatin capsule (i.e., the “digital pill”); a wireless, wearable RFID reader; and a secure, cloud-based online server and patient-facing smartphone app (Fig. 1) [14]. When a digital pill is ingested, the chloride ion gradient in the stomach activates the RFID tag, which emits a message containing time-stamped ingestion data to a wearable Reader device; the Reader can take several forms, including a lanyard-based device (as in the etectRx ID-Cap System) or a cutaneous patch worn on the abdomen (as in the Proteus Digital Health Feedback System) [15]. The Reader stores and transmits ingestion data via low energy Bluetooth to a patient’s smartphone, which permits on-demand user access to personal adherence data, as well as to an online dashboard accessible to the care team. Previous investigations have demonstrated the feasibility of utilizing a DPS to measure medication adherence in the context of multiple disease states, including hepatitis C, hypertension, and HIV pre-exposure prophylaxis [15, 16]. Prior studies conducted among TB patients in the US have demonstrated high DPS accuracy in detecting ingestion events and user acceptance of DPS technology that utilizes the cutaneous patch [16, 17].

Despite the demonstrated feasibility of DPS technology, however, it remains unclear whether the DPS can be implemented for ATT adherence monitoring in India, given key differences in the cultural context of adherence monitoring, availability of smartphone infrastructure, and a lack of documented perspectives of key stakeholders surrounding patient privacy in the context of TB and ATT. In this investigation, we conducted a quantitative assessment to understand the perspectives around DPS technology and willingness to utilize a DPS for ATT monitoring among clinical stakeholders involved in TB treatment in India. We additionally sought to understand how clinical ATT decisions surrounding dose adjustments, the intensity of adherence monitoring, and escalation of care in India could leverage real-time adherence data captured by the DPS.

## Methods

### Participants

A total of 51 adult participants enrolled in this study. Inclusion criteria included: (1) identified as clinicians or providers in India managing TB patients on ATT, and (2) reported more than 6 months of direct experience providing diagnosis, treatment, or ongoing monitoring of TB patients.

### Procedures

**Fig. 2:**
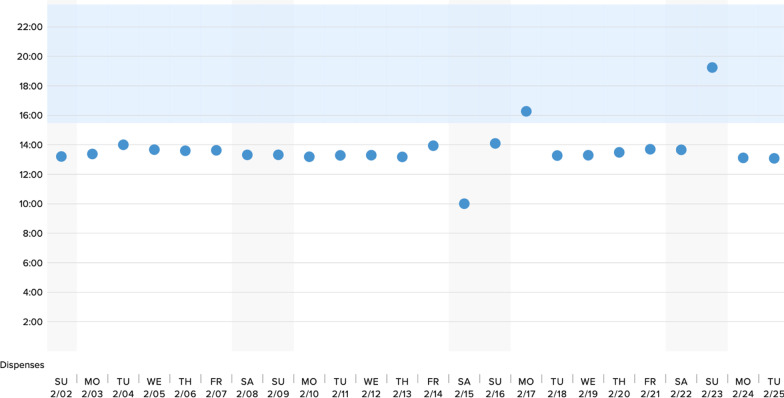
Example of DPS adherence data in context of continuous adherence

**Fig. 3:**
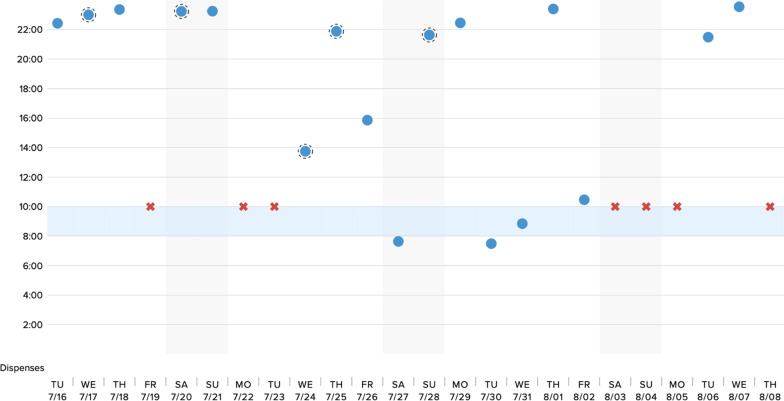
Example of DPS adherence data in context of suboptimal adherence

**Fig. 4:**
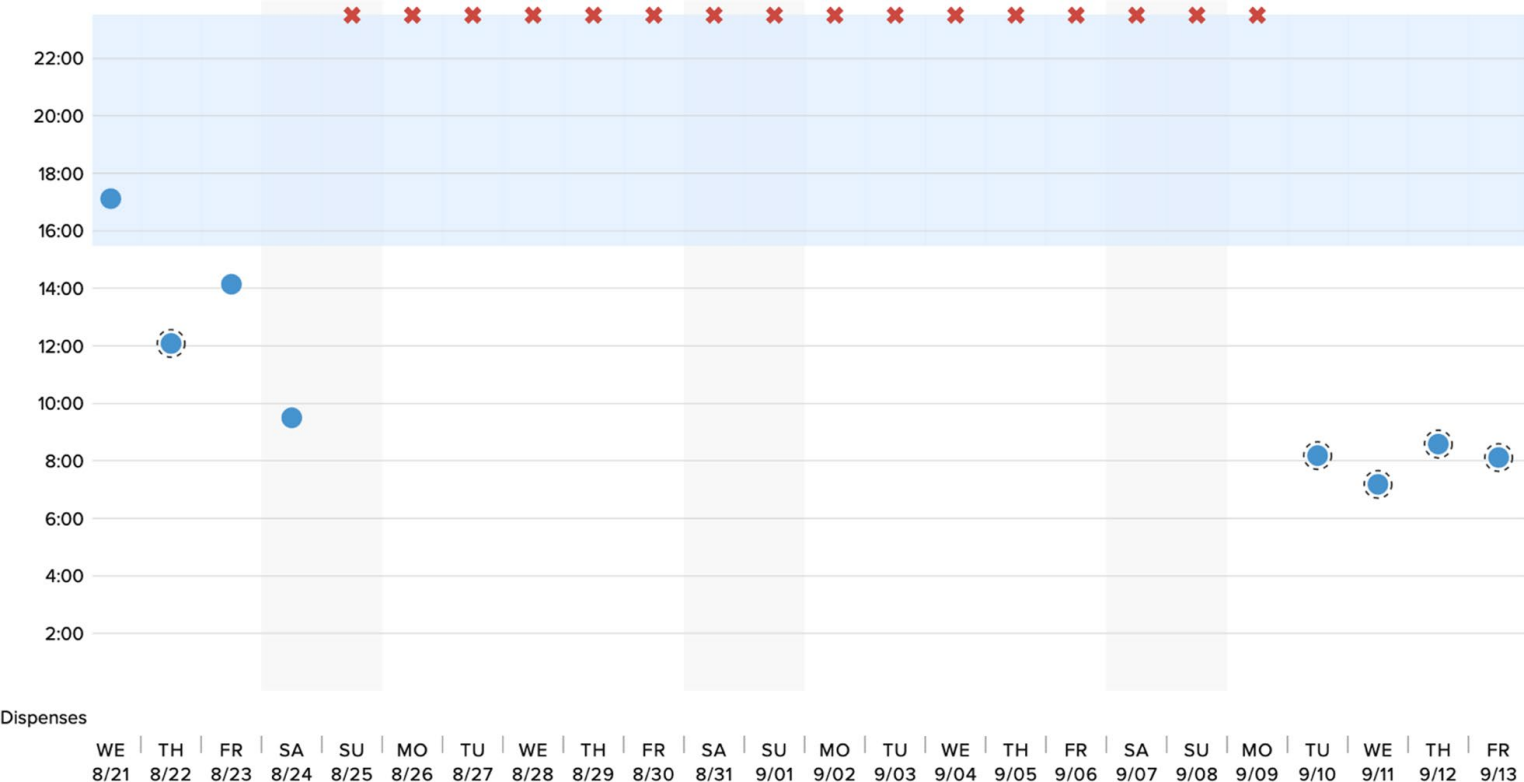
Example of DPS adherence data in context of frank nonadherence

Potential participants were recruited via personal contacts of the study team. We did not utilize a specific TB clinic. We obtained verbal informed consent from all study participants. This process was approved by our governing ethics board at Amala Institute of Medical Sciences, Thrissur, Kerala, India. Following enrollment, participants were provided with a link to a video describing the DPS technology and its functionality (Additional file 1). After viewing the video, participants completed a quantitative assessment, where they reported basic sociodemographic information, their degree of experience managing TB patients on ATT, perceived challenges surrounding ATT adherence, and previous adherence support tools they had recommended to their TB patients (Additional file 2). Participants also reported their initial impressions of the DPS technology, concerns regarding its functionality and deployment among patients in both the intensive and continuation phases of ATT, and perceived implementation challenges in India. Next, participants were presented with several examples of mock adherence data from the DPS, generated via the actual clinician-facing interface, which demonstrated (1) continuous adherence (i.e., ingestions recorded by the system daily), suboptimal adherence (i.e., ingestions sporadically missed) and frank nonadherence (i.e., lapses of missed doses for a week or more); these scenarios are illustrated in Figs. 2, 3, 4. They were asked to interpret each example of DPS-recorded adherence data (i.e., by assigning it to one of these three categories), and then to identify, from a list of possible treatment strategies, the interventions they would consider in each scenario (assuming that the theoretical patients were on Category 1 ATT treatment, i.e., new smear positive patients with pulmonary TB).

All study procedures were conducted between April 2021 and May 2022 in accordance with ethic guidelines of Amala Institute of Medical Sciences, Thrissur, Kerala, India. All methods were carried out in accordance with relevant guidelines and regulations. Ethical approval was obtained from the Institutional Ethics Committee at Amala Institute of Medical Sciences, Thrissur, Kerala, India.

### Data analysis

Basic means and standard deviations were calculated for sociodemographic variables and degree of practice experience to characterize the sample. Frequencies of response categories for survey questions were calculated. All analyses were conducted using Microsoft Excel.

## Results

A total of 73 individuals involved in the management of patients with TB were approached regarding participation in the study, and all met inclusion criteria. Fifty one provided verbal informed consent and enrolled. Twenty-two individuals declined to participate; reasons were not specified. One enrolled participant was ultimately excluded from the analyses, as they disclosed during enrollment that they were not actively managing patients with TB.

### Sociodemographics

The mean age was 34.3 years (SD = 7.3) and most participants (70%; N = 35) identified as male. The sample comprised internists (52%; N = 26), pulmonologists (30%; N = 15), National Tuberculosis Elimination Program (NTEP) officials (14%; N = 7), a WHO consultant (2%; N = 1), and a Revised National Tuberculosis Control Program (RNTCP) senior treatment supervisor (2%; N = 1). Most participants were from Kerala, India (84%; N = 42). Participants reported a median of 4 years’ experience in the management of TB patients (IQR 3, 6), with most of the sample (68%; N = 34) managing 50 or fewer TB patients during the past year. Almost all participants (98%; N = 49) reported discussing ATT adherence during clinical encounters, yet half (50%; N = 25) reported that less than 10% of their patients had difficulty with ATT adherence (Table 1).

**Table 1:** Sociodemographics and practice experience of sample (N = 50)

Variable	Mean (SD)
Age (in years)	34.3 (7.3)

	Median (IQR)
Years of experience in managing patients with TB	4 (3, 6)

	n (%)
Sex	
Male	35 (70%)
Female	15 (30%)
Occupational status	
General physician/practitioner (internist)	26 (52%)
Pulmonologist	15 (30%)
*NTEP officials	7 (14%)
WHO consultant	1 (2%)
**RNTCP Senior treatment supervisor	1 (2%)
Current clinical practice location	
Kerala	42 (84%)
Karnataka	1 (2%)
Madhya Pradesh	1 (2%)
Tamil Nadu	2 (4%)
Telangana	1 (2%)
Uttar Pradesh	1 (2%)
Not specified	2 (4%)
Number of patients with TB treated in the past year	
0–10	13 (26%)
11–50	21 (42%)
51–100	8 (16%)
More than 100	8 (16%)
Percentage of TB patients with perceived adherence issues	
Less than 10%	25 (50%)
10–25%	19 (38%)
26–50%	4 (8%)
51–75%	0 (0%)
76–100%	2 (4%)
Perceived reasons for nonadherence among patients on ATT	
Cost of transport to DOTS clinic	5 (10%)
Lack of understanding of importance of ATT adherence	38 (76%)
Stigma of TB	3 (6%)
Forgetfulness	9 (18%)
Improvement of symptoms	26 (52%)
Adverse drug effects	4 (8%)
Others	2 (4%)
Awareness of adherence monitoring strategies used for ATT	
99DOTS	33 (66%)
Regular DOTS	35 (70%)
Health worker’s visit at home	43 (86%)
Phone call or text message reminders	38 (76%)
Digital pill	10 (20%)
Digital pill box	7 (14%)
Other electronic adherence system	4 (8%)
Other	1 (2%)
Adherence monitoring strategies previously used with patients	
99DOTS	26 (52%)
Regular DOTS	29 (58%)
Health worker’s visit at home	34 (68%)
Phone call or text message reminders	19 (38%)
Digital pill	0
Digital pill box	0
Other electronic adherence system	2 (4%)
Other	2 (4%)
Asks patients about ATT adherence during clinical visits	
Yes	39 (78%)
No	11 (22%)

*NTEP: National Tuberculosis Elimination Program

**RNTCP: Revised National Tuberculosis Control Program

### Awareness of and willingness to use DPS

Most participants (86%; N = 43) indicated a willingness to use an adherence technology, such as a DPS, to measure medication adherence. Despite only one pilot randomized trial involving a DPS for the measurement of ATT adherence globally, some participants (22%; N = 11) reported an awareness of DPS technology prior to their enrollment in the study. After learning about the DPS from the video provided in the present study, more than three-quarters of participants (76%; N = 38) reported they would recommend the DPS to their TB patients as an adherence monitoring tool for ATT. Most (82%; N = 42) reported that the DPS would be directly applicable to ATT adherence monitoring—particularly given the decreased frequency of in-person DOT during the COVID-19 pandemic—and most (78%; N = 39) agreed that the DPS would be preferable to DOTS if its cost-effectiveness could be demonstrated. Nearly two-thirds of participants (64%; N = 32) reported that the DPS should be used for ATT adherence measurement during both the intensive and continuation phases of treatment. With respect to participants’ preferred frequency of access to DPS-recorded adherence data, (64%; N = 32) reported a desire to access and review data in real time. Finally, some participants (38%; N = 19) viewed the DPS as potentially intrusive to patients during the course of treatment, and the primary perceived barriers to DPS implementation were patient acceptance (92%; N = 46), cost (86%; N = 43), and a lack of infrastructure to support DPS use (66%; N = 33, Table 2).

**Table 2:** Awareness of and willingness to use digital pill system (DPS)

Variable	n (%)
Desire to use adherence technology to measure medication adherence	
Yes	43 (86%)
No	7 (14%)
Prior awareness of DPS technology	
Yes	11 (22%)
No	39 (78%)
Willing to recommend DPS to patients for ATT adherence monitoring	
Yes	38 (76%)
No	12 (24%)
Phases of ATT treatment in which DPS should be used for adherence monitoring	
Intensive phase*	8 (16%)
Continuation phase**	8 (16%)
Both phases	32 (64%)
Not useful for ATT	2 (4%)
Viewed DPS as intrusion into a patient’s life	
Yes	19 (38%)
No	31 (62%)
More likely to recommend DPS if cost-effective compared to DOTS	
Yes	39 (78%)
No	11 (22%)
Viewed DPS as a good alternative to DOTS in the context of COVID-19 pandemic	
Yes	41 (82%)
No	9 (18%)
Desire to see ATT adherence data from DPS in real time versus at regular clinical visits	
In real time	32 (64%)
At regular clinical visit	18 (36%)
Other perceived benefits of DPS	
Better drug adherence	39 (78%)
Improved insight into medication taking behavior	32 (64%)
Better physician–patient relationship	18 (36%)
Better knowledge on drug efficacy	18 (36%)
Early detection of drug toxicity	16 (32%)
Other	1 (2%)
Individuals who should have access to ATT adherence data from DPS	
District TB officer	23 (46%)
TB medical officer	33 (66%)
Treating physician	38 (76%)
Senior treatment supervisor—TB unit	20 (40%)
Social worker	22 (44%)
Patient	9 (18%)
Patient’s family members	12 (24%)
Type of patients on ATT who would benefit from use of DPS	
All patients	12 (24%)
Individuals with risk of nonadherence	32 (64%)
Individuals with multi-drug resistant TB	26 (52%)
Individuals with demonstrated nonadherence to ATT	32 (64%)
Individuals with HIV	19 (38%)
Individuals with substance use disorders	17 (34%)
Perceived barriers to DPS implementation	
Patient acceptance of DPS	46 (92%)
Provider acceptance of DPS	24 (48%)
Lack of infrastructure to support DPS	33 (66%)
Cost	43 (86%)
Increased workload for provider	12 (24%)

*Intensive Phase—First 8 weeks of the drugs Isoniazid (H), Rifampicin (R), Pyrazinamide (Z) and Ethambutol (E)

**Continuation Phase—Drugs Isoniazid, Rifampicin and Ethambutol given for another 16 weeks after the Intensive Phase

### ATT treatment decisions informed by DPS data

Participants were presented with three examples of mock DPS adherence data intended to illustrate (1) continuous adherence, (2) suboptimal adherence, and (3) frank nonadherence (Figs. 2, 3, 4). Participants were able to accurately identify patterns of continuous adherence (80%; N = 40), suboptimal adherence (90%; N = 45), and frank nonadherence (82%; N = 41). In the scenario where adherence was continuous, 54% (N = 27) of participants reported they would continue ATT monitoring, while 32% (N = 16) reported that they would utilize DPS data to reinforce adherence behaviors (Fig. 2, Table 3).

**Table 3:** Interpretation of DPS data and proposed ATT treatment decisions in context of continuous adherence

Variable	n (%)
Interpretation of adherence category	
Fully adherent	40 (80%)
Partially/suboptimally adherent	10 (20%)
Nonadherent	0 (0%)
ATT treatment decisions informed by DPS data	
No action (continued monitoring)	27 (54%)
Reinforce adherence through counseling at clinical visit	16 (32%)
Phone call to patient	8 (16%)
Health care worker visit	6 (12%)
Transition to DOTS	1 (2%)
Test for multidrug resistant TB (MDR-TB)	1 (2%)
Other	1 (2%)

In the suboptimal adherence scenario, participants reported that they would reinforce adherence through counseling at a clinical visit (58%; N = 29), calling the patient directly to discuss ATT adherence (46%; N = 23), or asking a health care worker to visit the patient (48%; N = 24). Some participants additionally reported that suboptimal adherence detected by the DPS would prompt them to consider a transition to DOTS (24%; N = 12, Fig. 3, Table 4).

**Table 4:** Interpretation of DPS data and proposed ATT treatment decisions in context of suboptimal adherence

Variable	n (%)
Interpretation of adherence category	
Fully adherent	2 (4%)
Partially/suboptimally adherent	45 (90%)
Nonadherent	3 (6%)
ATT treatment decisions informed by DPS data	
No action (continued monitoring)	0 (0%)
Reinforce adherence through counseling at clinical visit	29 (58%)
Phone call to patient	23 (46%)
Health care worker visit	24 (48%)
Transition to DOTS	12 (24%)
Test for multidrug resistant TB (MDR-TB)	4 (8%)
Other	2 (4%)

Finally, in the frank nonadherence scenario, participants indicated that they would use DPS data to reinforce ATT adherence via counseling at a clinical visit (44%; N = 22), calling the patient to discuss their adherence (46%; N = 23), and asking a health care worker to visit the patient to understand reasons for nonadherence (58%; N = 29). Faced with DPS-detected nonadherence, a majority of participants (60%; N = 30) indicated they would transition their patients to DOTS, and nearly one-third (30%; N = 15) would consider testing for MDR-TB (Fig. 4, Table 5).

**Table 5:** Interpretation of DPS data and proposed ATT treatment decisions in context of frank nonadherence

Variable	n (%)
Interpretation of adherence category	
Fully adherent	0 (0%)
Partially/suboptimally adherent	8 (16%)
Nonadherent	82 (42%)
ATT treatment decisions informed by DPS data	
No action (continued monitoring)	0 (0%)
Reinforce adherence through counseling at clinical visit	22 (44%)
Phone call to patient	23 (46%)
Health care worker visit	29 (58%)
Transition to DOTS	30 (60%)
Test for multidrug resistant TB (MDR-TB)	15 (30%)
Other	5 (10%)

## Discussion

Challenges to adherence to ATT are multifactorial and complex, encompassing structural factors (e.g., access to medications, transportation, cost), behavioral factors (e.g., comorbidities that may be associated with nonadherence), and medical factors (e.g., lack of perceived clinical efficacy, adverse events) [18–20]. Adherence supports can be employed to address one or more of these factors; as in other diseases, the ultimate goal of such approaches is to reinforce adherence behavior and to provide tools that empower individuals and their care teams to maintain ATT adherence and prevent disease transmission. Unlike other adherence measurement systems, the DPS offers potential benefit by directly confirming medication ingestions and providing a platform for adherence supports that may aid in addressing barriers to adherence. This investigation demonstrates that clinicians prescribing ATT in southern India are accepting of DPS technology and find it applicable among their current ATT protocols. Key barriers to DPS implementation in this context were also identified and will need to be addressed in parallel with DPS use in India. Finally, this study demonstrates that ATT adherence data recorded by the DPS can be utilized by clinicians to make management decisions relevant to the course of TB treatment.

Our results indicated that a majority of participants (86%) were accepting of the DPS for the measurement of ATT adherence. The DPS was novel to most of the sample (78%), and many viewed the system as innovative and applicable for ATT treatment in their personal practices. Participants identified key groups who would benefit most from the DPS, including those with prior adherence challenges and those with MDR-TB. For such individuals, participants perceived the DPS to be useful for adherence monitoring during both the initiation and continuation phases of ATT. In light of the COVID-19 pandemic, the DPS was additionally seen as a potential alternative that could function as a surrogate of traditional DOTS, given its capacity to directly confirm ingestion events remotely [19]. These data indicate that, from a provider perspective, the DPS is an attractive tool for measuring ATT adherence; however, participants did acknowledge several potential barriers to DPS implementation in India. Specifically, the overall healthcare costs incurred by the deployment of the DPS has yet to be measured in India and compared to that of other technology-mediated adherence support tools. Key among measuring these costs will be the price of hardware (e.g. digital pills and data support infrastructure) as well as person power (for maintenance, interpretation of data). While activation costs of these systems may be high, previous work in other countries suggests that a gradual scale-up of technology and personnel infrastructure needed to manage these systems may ultimately make them cost effective in the long term [21, 22]. Despite these concerns, earlier work has demonstrated that the costs of deploying a DPS for TB ATT adherence may ultimately be cost effective when considering costs associated with monitoring and managing DOT therapy in both WHO recommended seven and 3 day DOT models [23]. Additionally, from a clinician perspective, patient acceptance of the DPS and willingness to operate this technology were identified as key potential barriers that may impact adoption of a DPS for ATT in this context. Previous pilot data from individuals with TB using ingestible sensor systems for ATT in the United States demonstrate high patient acceptance of the system and a desire to remain on DPS-based ATT adherence monitoring compared to DOT [16]. These sentiments mirror our previous work in HIV pre-exposure chemoprophylaxis that demonstrates high patient acceptance of these systems [24–26]. We anticipate that prior to deployment in India, patient perspectives of the DPS should be explored.

This study also demonstrated that ATT prescribers were able to utilize sample DPS-recorded adherence data to make clinical decisions around treatment course for their TB patients. Participants were able to successfully interpret patterns of continuous adherence, suboptimal adherence, and frank nonadherence. These adherence patterns informed participants’ proposed plans for subsequent clinical interventions, which included encouraging adherence, scaling up in-person adherence support, and transitioning from a DPS-based adherence program to DOTS. Frank nonadherence in particular triggered participants to report that they would consider testing such patients for the development of MDR-TB. These data suggest that clinicians view the DPS as a modality that would allow them to better understand their patients’ individual ATT adherence patterns in real time, which could then be used as a basis for the delivery of personalized adherence interventions. This study demonstrates that clinicians see value in the system, and that DPS-recorded adherence data is readily accessible and understood in the continuum of typical TB care.

Overall, these findings indicate that a DPS is perceived as an acceptable and useful technology among clinicians in India who manage patients with TB. Most clinicians are aware of adherence technologies (e.g., DOTS, 99DOTS, healthcare worker visit and mobile phone reminders) and utilize them in their practice. Deployment of a DPS may therefore be a reasonable next step to pursue in selected patients with TB. Given acceptance by clinicians and their ability to successfully interpret DPS adherence data, future work should focus on understanding responses to DPS technology and use of the DPS as an adherence tool in the real world among TB patients themselves, as well as among patient advocates and TB community advisory boards—all of whom may have different attitudes toward DPS than providers. DPS-based adherence monitoring could potentially be used as an independent DOT tool, or may be investigated to enable wider adoption of other strategies like self-administered ATT and family DOT. Additionally, given the finding that clinicians can use DPS data to make management decisions around ATT adherence monitoring, future research should explore the specification and design of ATT interventions that are built around real-time DPS adherence data. While previous work has developed smartphone-based reminder systems and personalized adherence interventions that are delivered in response to DPS data for other diseases (e.g., HIV pre-exposure prophylaxis), researchers should incorporate key cultural underpinnings of adherence monitoring in India, as well as mobile phone literacy, which may define both the platform and content of ATT adherence interventions in India [27, 28].

There are several limitations in this study. First, due to the pilot nature of this study, the sample was small and primarily located in Kerala, India. Individuals practicing in Kerala may have different perspectives around DPS technology, as well as different degrees of access to technology and experience with TB management, as compared to those in other regions of India; therefore, additional research is needed to evaluate the perspectives of providers involved in TB care across different regions of India. Second, we conducted quantitative assessments without actual deployment of the DPS. While participants reported general acceptance of the technology following a description and educational video outlining its functionality, acceptance of the DPS may vary in the setting of actual use. Third, this investigation assessed perceptions of the DPS among providers involved in the management of TB only; future work should focus on exploring perceptions of the DPS from the perspective of patients, patient advocates, and community advisory board members. These providers were predominantly male which do not reflect the demographic of TB providers throughout India. Future work should seek to understand perceptions of the DPS from female providers managing patients with TB. Despite these limitations, this investigation is the first to explore the acceptance and potential application of a DPS for ATT adherence monitoring in India, which may inform future work to justify additional DPS-based research in India and similar settings.

## Conclusions

In this study of stakeholders directly involved in TB management in India, DPS technology was viewed as an acceptable, feasible, and useful tool for monitoring ATT adherence in India. Future research should explore patient acceptance of the DPS and pilot demonstrations of the technology among TB patients on ATT in this context.

## Supplementary Information

**Additional file 1.** Recorded video demonstrating the functionality of the digital pill system used as part of the quantitative assessment.

**Additional file 2.** Quantitative assessment administered to study participants.

## Data Availability

The datasets used and analyzed during the current study are available from the corresponding author on reasonable request.

## Abbreviations

TB: Tuberculosis
MDR: TB—multi-drug resistant tuberculosis
HIV: Human Immunodeficiency Virus
CDC: Center for disease control and prevention
WHO: World Health Organization
ATT: Antitubercular therapy
DOTS: Directly observed therapy—short course
VDOT: Video DOT
MERM: Medication Event Reminder Monitor
DPS: Digital pill system
RFID: Radiofrequency identification
NTEP: National Tuberculosis Elimination Program
RNTCP: Revised National Tuberculosis Control Program
