# Supplementary material for: Formative acceptance of ingestible biosensors to measure adherence to TB medications
Source: BMC Infect Dis. 2022 Sep 28;22:754. doi: 10.1186/s12879-022-07756-x (PMC9517983; doi:10.1186/s12879-022-07756-x)
Supplement: Supplementary file 1 — Additional file 1. Recorded video demonstrating the functionality of the digital pill system used as part of the quantitative assessment. [file 12879_2022_7756_MOESM1_ESM.docx]

YouTube link to DPS information video:

<https://youtu.be/Wgw2VH8P2z8>
