# Supplementary material for: Formative acceptance of ingestible biosensors to measure adherence to TB medications
Source: BMC Infect Dis. 2022 Sep 28;22:754. doi: 10.1186/s12879-022-07756-x (PMC9517983; doi:10.1186/s12879-022-07756-x)
Supplement: Supplementary file 2 — Additional file 2. Quantitative assessment administered to study participants. [file 12879_2022_7756_MOESM2_ESM.docx]

Supplementary material 2: Quantitative assessment

Thank you for your participation in the study

1. What is your age in years?
2. What is your gender?

A) Male
B) Female

C) Other

1. What is your designation/occupation in TB treatment?
2. State TB officer
3. District TB Officer
4. WHO consultant
5. General physician
6. Pulmonologist
7. General practitioner
8. NTEP nodal officer
9. NTEP MO
10. Senior treatment supervisor
11. TB health visitor
12. Other
13. What is your state of residence in India?
14. How many years of experience do you have in TB program?
15. How many TB patients have you treated in the past year?
16. 0 – 10
17. 11 – 50
18. 51 – 100
19. >100
20. Not Applicable
21. What percentage of your patients do you think have adherence issues?
22. <10%
23. 10-25%
24. 26 - 50%
25. 51-75%
26. 76-100%
27. Do you ask about ATT adherence during clinical visits?
28. Yes
29. No
30. Not applicable
31. What are some of the reasons in your opinion for patients to be non-adherent to their TB treatment? (Select all that apply)
32. Cost of transport to DOTS clinic
33. Lack of understanding of the importance of ATT adherence
34. Stigma of TB
35. Forgetfulness
36. Improvement of symptoms
37. Other
38. Would you want to use an adherence technology to measure medication adherence?
39. Yes
40. No
41. What adherence strategies for ATT have you heard of? (Select all that apply)
42. 99 DOTS
43. Regular DOTS
44. Health workers' visit
45. Phone call or text message reminders
46. Digital pill
47. Digital pill box
48. Other electronic adherence system
49. Other: ___________
50. Have you heard of the digital pill system or ingestible sensors before?
51. Yes
52. No
53. What adherence strategies for ATT have you used in your patients? (Select all that apply)
54. 99 DOTS
55. Regular DOTS
56. Health workers' visit
57. Phone call or text message reminders
58. Digital pill
59. Digital pill box
60. Other electronic adherence system
61. Other
62. If a digital pill system for ATT adherence monitoring were to be used in India, which phase would be best to implement this technology?
63. Intensive phase
64. Continuation phase
65. Both phases
66. Not useful for ATT
67. What other benefits might the digital pill system have? (Select all that apply)
68. Better drug adherence
69. Improved insight into medication taking behavior
70. Better Physician Patient relationship
71. Better knowledge on drug efficacy
72. Early detection of Drug toxicity
73. Other
74. If the digital pill were an option, would you recommend your patients use it for monitoring ATT adherence?
75. Yes
76. No
77. What are your concerns if we introduce the digital pill system for the monitoring of ATT instead of DOTS? (Select all that apply)
78. Safety
79. Privacy
80. Acceptance
81. Cost effectiveness
82. Other
83. Do you think the digital pill system used for monitoring ATT adherence will be an intrusion into a patient’s life?
84. Yes
85. No
86. If the digital pill system is cheaper than the DOTS program, would you prefer to recommend it?
87. Yes
88. No
89. In your opinion do you think the digital pill system is a good alternative for DOTS considering the current COVID 19 pandemic?
90. Yes
91. No
92. For the next two questions, please refer to the following graphs of adherence. Imagine that these are adherence patterns you receive from a patient of yours in the DOTS clinic on ATT who is initiating TB treatment for Category I TB. Blue circles demonstrate medication ingestion events, red X marks days when no ingestion event was recorded.

Patient A


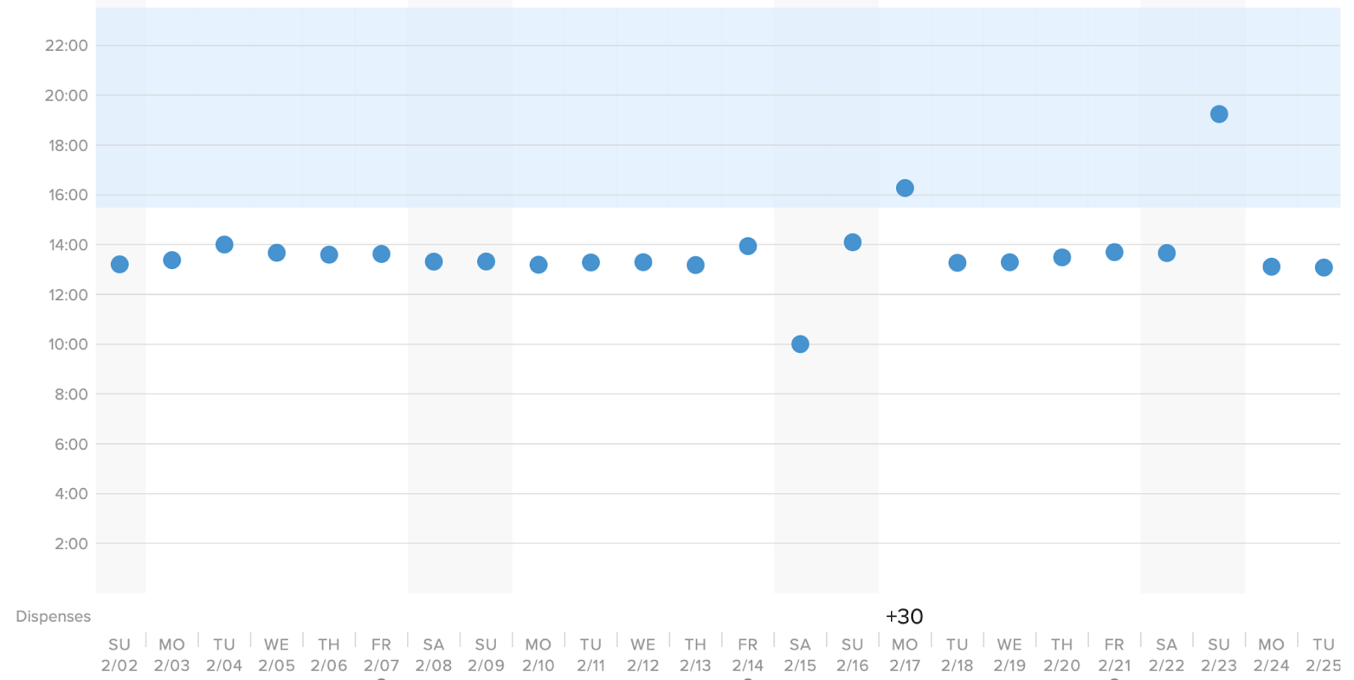


Patient B


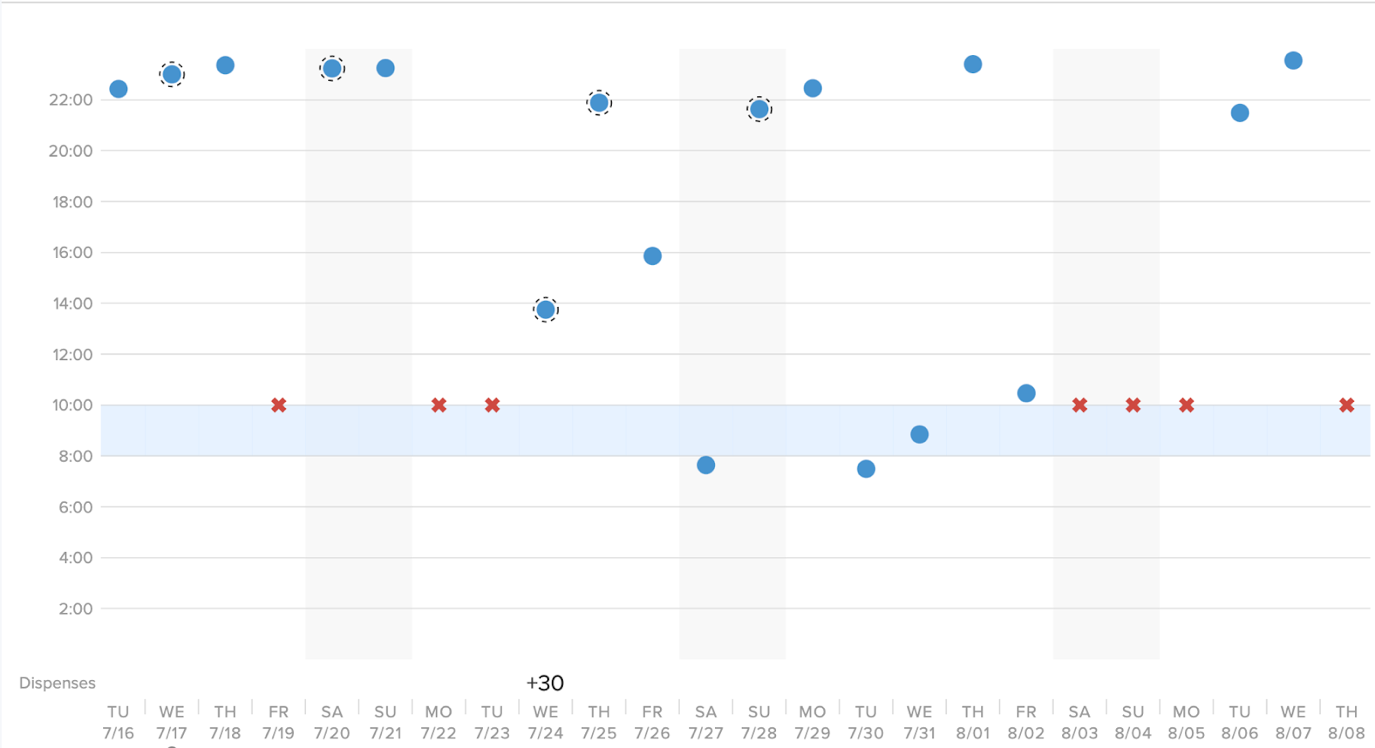


Patient C


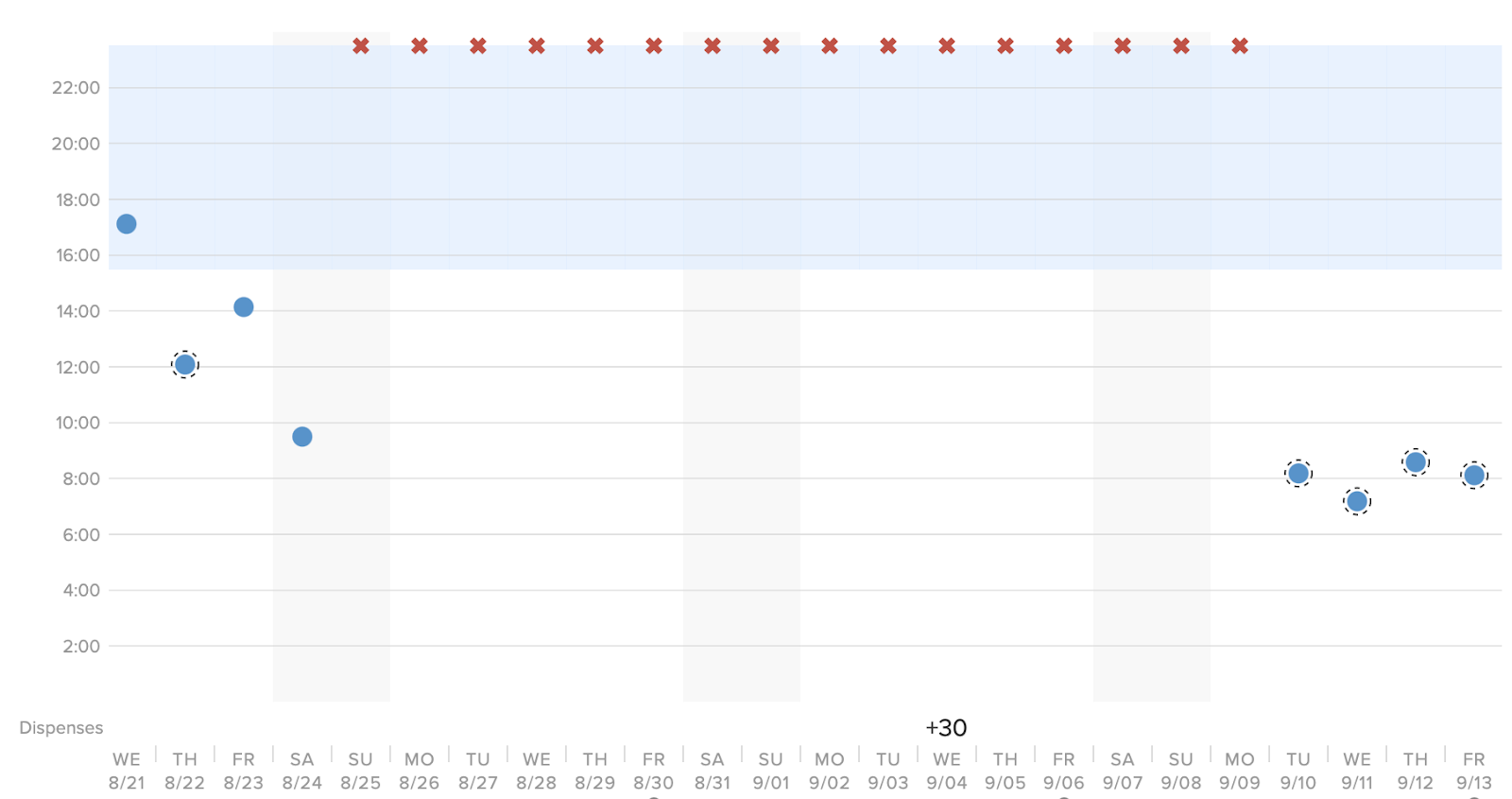


Looking at adherence data above, tell us how you would rate the adherence of each patient (A, B, and C):

| Fully adherent Partially/suboptimal adherence Nonadherent |
| --- |
| A |
| B |
| C |

For each of the patients, how would you manage them if you were given access to this adherence data at the DOTS clinic? (Select all that apply)

|  | **A** | **B** | **C** |
| --- | --- | --- | --- |
| No action |  |  |  |
| Reinforce adherence through counselling |  |  |  |
| Phone call to patient |  |  |  |
| Health worker visit |  |  |  |
| Transition to DOTS |  |  |  |
| Test for drug resistant TB |  |  |  |
| Others |  |  |  |

1. Who should be the primary person receiving adherence data from the digital pill system?
2. District TB office
3. TB medical officer
4. Treating Physician
5. Senior treatment supervisor
6. TB health visitor
7. Patient
8. Patient’s family members
9. Other
10. Who should have access to adherence data from the digital pill? (Select all that apply)
11. District TB office
12. TB medical officer
13. Treating Physician
14. Senior treatment supervisor
15. TB health visitor
16. Patient
17. Patient’s family members
18. Other
19. Which type of patients on ATT do you think digital pills should be used in? (Select all that apply)
20. All patients
21. Individuals with risk of non-adherence
22. Individuals who have multi-drug resistant TB
23. Individuals who have demonstrated non-adherence to ATT
24. Individuals with HIV
25. Individuals with substance use disorders
26. Other
27. If you had such a system, would you want to see the data in real-time or at regular clinical visits?
28. Yes, I would like to see the data Real time
29. No, I would like to see the data at regular clinical visit
30. What do you think are some of the challenges that we may face in the implementation of such a system? (Select all that apply)
31. Cost
32. Willingness of patients to accept the system
33. Willingness of providers to use the system
34. Infrastructure to support digital pills (pharmacy etc)
35. Increased workload for the provider
36. Other
